# Localisation pattern of Foxp3^+^ regulatory T cells is associated with clinical behaviour in gastric cancer

**DOI:** 10.1038/sj.bjc.6604149

**Published:** 2007-12-18

**Authors:** Y Mizukami, K Kono, Y Kawaguchi, H Akaike, K Kamimura, H Sugai, H Fujii

**Affiliations:** 1 First Department of Surgery, University of Yamanashi, 1110 Shimokato, Chuo-City, Yamanashi 409-3898, Japan

**Keywords:** Foxp3, regulatory T cells, gastric cancer, prognosis

## Abstract

It has been reported that the population of regulatory T cells (T regs) is increased in tumour-infiltrating lymphocytes in cancer-bearing hosts. Recently, forkhead/winged helix transcription factor p3, Foxp3, is thought to be the most reliable marker of T regs. In the present study, we investigated the prevalence and localisation pattern of Foxp3^+^ cells in gastric cancer (*n*=80) by immunohistochemistry, in relation to the clinical outcome of gastric cancer patients. Immunohistochemical staining was performed with anti-Foxp3 mAb, and Foxp3^+^ cells were semiquantified. We divided all cases into two groups: Foxp3^+^-high (*n*=40) and Foxp3^+^-low (*n*=40) groups, by the median size of the population of Foxp3^+^ cells. Furthermore, in terms of the localisation pattern of accumulating Foxp3^+^ cells in tumours, we classified all cases into three groups: a peri-tumour group (*n*=30), a diffuse group (*n*=40), and a follicular group (*n*=10). As a result, although the populations of Foxp3^+^ cells in stage IV were significantly larger than those in stage I (*P*<0.05), there was no significant difference in survival between the patients with high and low population levels of Foxp3^+^ cells. However, survival in patients with a diffuse pattern of Foxp3^+^ cells was significantly poorer than in those with a peri-tumoral pattern. In conclusion, the localisation pattern, but not the population size, of Foxp3^+^ cells was significantly related to patient survival.

Regulatory T cells (T regs) are one of the T-cell subsets, which play important roles in immunological self-tolerance (Sakaguchi *et al*, 1995; Jonuleit *et al*, 2001; Ng *et al*, 2001; Beyer and Schultze, 2006). They have a functionally immunosuppressive property that inhibits effector cells from acting against self in autoimmune diseases or a tumour (Sakaguchi *et al*, 1995; Jonuleit *et al*, 2001; Ng *et al*, 2001; Beyer and Schultze, 2006). They constitutively express CD25 (IL-2 receptor *α*-chain), CD45RO, glucocorticoid-induced tumour-necrosis factor receptor-related protein, and cytotoxic T-lymphocyte-associated antigen-4 (Sakaguchi *et al*, 1995; Dieckmann *et al*, 2001; Beyer and Schultze, 2006). Recently, it has been reported that Foxp3, forkhead/winged helix transcription factor, is a reliable marker of T regs (Hori and Sakaguchi, 2004; Yagi *et al*, 2004). Therefore, it is possible to define T regs more strictly as CD4^+^CD25^+^Foxp3^+^ cells.

In mice, it is known that autoimmune diseases, such as ulcerative colitis or Crohn's disease, occur due to the depletion of T regs (Hori and Sakaguchi, 2004; Sakaguchi *et al*, 2006). Also in humans, immune dysregulation polyendocrinopathy enteropathy X-linked syndrome is an autoimmune disease due to a deficiency of T regs (Kelsen *et al*, 2005; Dejaco *et al*, 2006; Sakaguchi *et al*, 2006; Takahashi *et al*, 2006). These observations indicated that T regs play important roles in immunological homeostasis. Although the mechanisms of suppression by T regs are still unclear, it has been reported that T regs can inhibit the function of effector T cells directly through cell-to-cell contact or indirectly via the secretion of immune-suppressive cytokines, and also suppress the Ag-presenting function of dendritic or NK cells (Dieckmann *et al*, 2002; Misra *et al*, 2004; Earle *et al*, 2005; Longhi *et al*, 2006; Smyth *et al*, 2006).

Recently, many studies in murine models have shown that the depletion of T regs can amplify antitumour immunity (Viehl *et al*, 2006; Rudge *et al*, 2007). Moreover, in humans, many studies have revealed that the population of T regs in tumour-infiltrating lymphocytes (TILs) is significantly larger than in normal tissues in several malignancies (Woo *et al*, 2001; Liyanage *et al*, 2002; Ormandy *et al*, 2005; Badoual *et al*, 2006; Petersen *et al*, 2006). We have reported that the frequency of T regs among TILs, tumour-draining regional lymph nodes, and peripheral blood lymphocytes is higher in gastric and oesophageal cancer patients than that in their normal counterparts (Ichihara *et al*, 2003). Importantly, after patients received curative resections of gastric cancers, the increased proportion of T regs was significantly reduced, and the levels were almost equal to those in normal healthy donors (Kono *et al*, 2006). These results strongly suggest that tumour-related factors induce and expand T regs. There is, however, still limited information describing the mechanisms behind T reg accumulation within cancer microenvironments and their expansion locoregionally. Thus, it is important to evaluate the localisation of infiltrating T regs in relation to the clinical outcome.

In the current study, we investigated the population and localisation pattern of Foxp3^+^ T regs in gastric cancer by immunohistochemistry, and evaluated the relationship between the findings and clinical outcome.

## MATERIALS AND METHODS

### Patients and samples

Eighty patients with gastric cancer, who were operated on in the University of Yamanashi Hospital from 1997 to 1998, were enrolled in the present study. The median follow-up time of the patients was 87.7 months. The characteristics of the study subjects are summarised in Table 1. None of the patients received radiotherapy, chemotherapy, or other medical interventions before the study. This study was approved by the Ethical Committee of the University of Yamanashi, and written informed consent was obtained from all individuals.

### Immunohistochemical analysis

Foxp3 staining was conducted using the avidin–biotin–peroxidase complex method with paraffin-embedded, 4-*μ*m-thick sections of gastric cancer. Briefly, each paraffin section was dewaxed, followed by antigen retrieval with Epitope Retrieval Solution (10 mmol citrate buffer (pH 6.0), Dakocytomation, Glostrup, Denmark) in a preheated water bath (98°C, 40 min), and endogenous peroxidase was blocked by ChemMate Peroxidase Blocking Solution (Dako). Then, biotinylated anti-human Foxp3 antibody (diluted by PBS, 1 : 20; eBioscience, San Diego, CA, USA) was applied for 40 min at room temperature. Thereafter, the sections were incubated with streptavidin-conjugated horseradish peroxidase (Dako) for 10 min, followed by development with 3,3′-diaminobenzidine (Dako) for 5 min and counter-staining with haematoxylin. Negative control staining was performed with isotype control, mouse IgG2a (Dako), instead of the primary antibody.

Quantitative evaluation of Foxp3^+^ cells was analysed in five randomly selected areas at a magnification of × 400 by two observers (YM and KK) in a blinded manner. Then, all cases with gastric cancer were divided into two groups: Foxp3^+^-high and Foxp3^+^-low groups, classified by the median value of the total population of Foxp3^+^ cells (median=34.5, range=2–525).

Furthermore, localisation patterns of infiltrating Foxp3^+^ cells in the tumour were divided into three groups: a peri-tumour group, a diffuse group, and a follicular group. The peri-tumour group was defined as the population of Foxp3^+^ cells in the peri-tumoral region that had increased more than five-fold compared to that in the central region of the tumour at a magnification of × 400, while the diffuse group was defined as the difference in the Foxp3^+^ cells between the peri-tumoral region and central region of the tumour that was less than five-fold. Moreover, the follicular group was defined as the population of Foxp3^+^ cells that mainly occupied the lymphoid follicles of the submucosal layer compared to any other region of the tumour.

### Statistical analysis

Actuarial overall survival rates were analysed by the Kaplan–Meier method, and survival was measured in days from the operation to death or the last review. Differences between survival curves were analysed by the log-rank test. Deviation in immunohistochemical patterns was evaluated by the *χ*^2^ test.

To assess the correlation between survival time and multiple clinicopathologic variables, univariate and multivariate analyses were conducted using Cox's proportional hazards model. Differences were considered significant at *P*<0.05. All statistical analyses were performed with StatView-J 5.0 software (Abacus Concepts, Berkeley, CA, USA).

## RESULTS

### Immunohistochemical analysis of Foxp3^+^ cells in gastric cancer

**Figure 1:**
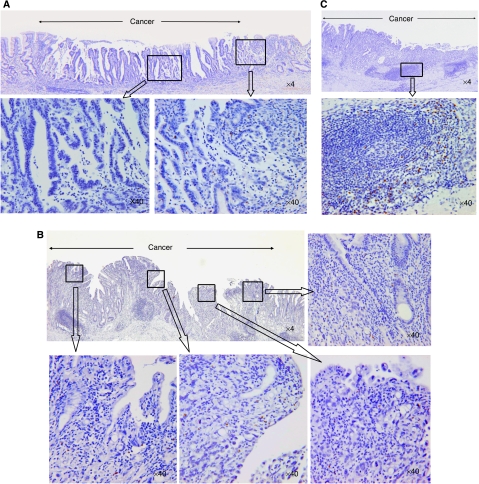
Localisation pattern of accumulating Foxp3^+^ cells in gastric cancer by immunohistochemistry. Localisation patterns of infiltrating Foxp3^+^ cells in the tumour were divided into three groups: a peri-tumour group (**A**, *n*=30), a diffuse group (**B**, *n*=40), and a follicular group (**C**, *n*=10). Representative staining is demonstrated. The peri-tumour group was defined as the population of Foxp3^+^ cells in the peri-tumoral region that had increased more than five-fold compared to that in the central region of the tumour at a magnification of × 400, while the diffuse group was defined as the difference in the Foxp3^+^ cells between the peri-tumoral region and central region of the tumour that was less than five-fold. The follicular group was defined as the population of Foxp3^+^ cells that mainly occupied lymphoid follicles of the submucosal layer compared to any other region of the tumour (original magnification: × 4, × 40).

Foxp3^+^ cells were specifically identified and semiquantified by immunohistochemistry (Figure 1A–C). Then, localisation patterns of infiltrating Foxp3^+^ cells were divided into three groups: a peri-tumour group (Figure 1A, *n*=30), diffuse group (Figure 1B, *n*=40), and follicular group (Figure 1C, *n*=10), in terms of where Foxp3^+^ cells dominantly occupied the lesion by immunohistochemistry as described in Materials and Methods. There was a very small population of Foxp3^+^ cells in normal gastric mucosa in the same specimens (Table 2). The population of Foxp3^+^ cells in stage IV was significantly larger than those in stage I (Table 2, *P*<0.05). There were no significant differences in the prevalence of Foxp3^+^ cells among T factors, N factors, or between histological classifications (Table 2).

Interestingly, tumours with a diffuse distribution pattern of Foxp3^+^ cells were more frequent in stages II+III+IV, while tumours with a peri-tumoral distribution pattern of Foxp3^+^ cells were more frequent in stage I (*P*<0.05 by the *χ*^2^ test, Table 2). There were no significant differences in the localisation pattern of Foxp3^+^ cells among T factors or between histological classifications, although there was significant difference in the localisation pattern of Foxp3^+^ cells among N factors (Table 2).

### The frequency and distribution pattern of Foxp3^+^ cells relating to survival in gastric cancer

**Figure 2:**
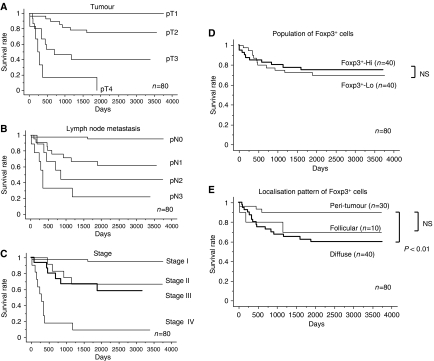
The localisation pattern, but not the population size, of Foxp3^+^ cells was significantly related to patient survival. The median survival time in early disease was significantly longer than that in advanced disease in terms of the pathologic tumour status (**A**), pathologic node status (**B**), and stage (**C**) (*n*=80, *P*<0.05, by the log-rank test). (**D**) Foxp3^+^-high and Foxp3^+^-low groups were classified by the median value of the population of Foxp3^+^ cells (median value=34.5, range=2–525). NS: not significant. (**E**) Localisation patterns of infiltrating Foxp3^+^ cells in the tumour were divided into three groups: a peri-tumour group (*n*=30), a diffuse group (*n*=40), and a follicular group (*n*=10).

While the grade of the T factor (Figure 2A), N factor (Figure 2B), and stage classification (Figure 2C) was significantly correlated with the survival of patients, there was no significant difference in survival between patients with large and small populations of Foxp3^+^ cells (Figure 2D). However, the survival rate in patients with a diffuse pattern of Foxp3^+^ cells was significantly poorer than in those with a peri-tumoral pattern (Figure 2E). These results indicated that the localisation pattern, but not the population size, of Foxp3^+^ cells was significantly related to patient survival.

To further assess whether the localisation pattern of Foxp3^+^ cells represented a prognostic parameter, we used Cox's proportional hazards model. The covariate parameters included several clinicopathologic factors, as shown in Table 3. On univariate analysis, a diffuse group in terms of the localisation of Foxp3^+^ cells showed a significantly higher hazard ratio for a poor prognosis (*vs* the peri-tumoral group, hazard ratio=4.65 (1.35–15.96), *P*<0.02), although multivariate analysis revealed that the localisation pattern of Foxp3^+^ cells was not an independent prognostic factor (*P*=0.39, Table 3).

## DISCUSSION

In general, T regs have functionally suppressive actions on other effector T cells (Sakaguchi *et al*, 1995; Dieckmann *et al*, 2001; Jonuleit *et al*, 2001; Ng *et al*, 2001; Earle *et al*, 2005; Beyer and Schultze, 2006). They have been characterised as a CD4^+^CD25^high^ population among CD4^+^ T cells (Sakaguchi *et al*, 1995; Dieckmann *et al*, 2001; Jonuleit *et al*, 2001; Ng *et al*, 2001; Earle *et al*, 2005; Beyer and Schultze, 2006). However, it was difficult to discriminate T regs from conventional effector T cells that express CD25 intermediately, because effector T cells continuously express CD25 on their cell surface (Zheng *et al*, 2004; Fontenot *et al*, 2005; Allan *et al*, 2007; Pillai *et al*, 2007). Recently, it has been reported that Foxp3, forkhead/winged helix transcription factor, is the most reliable marker of T regs (Hori and Sakaguchi, 2004; Yagi *et al*, 2004; Fontenot *et al*, 2005). Therefore, it is possible to define T regs more strictly as CD4^+^CD25^+^Foxp3^+^ cells. Moreover, Foxp3 expression is crucial regarding whether the cells have a virtually suppressive function. Thus, Foxp3^+^ cells were analysed by immunohistochemistry in order to evaluate T regs in gastric cancer.

Previous studies in human malignancies reported that the prevalence of T regs defined as a CD4^+^CD25^+^ population was significantly increased in TILs compared to the normal counterparts (Woo *et al*, 2001; Liyanage *et al*, 2002; Ormandy *et al*, 2005; Badoual *et al*, 2006; Petersen *et al*, 2006). Furthermore, the high prevalence of CD4^+^CD25^+^ T regs is closely associated with a poor prognosis in ovarian (Curiel *et al*, 2004) or pancreatic (Liyanage *et al*, 2002) cancer. Recently, intratumoral Foxp3^+^ T regs in ovarian (Wolf *et al*, 2005) or pancreatic (Hiraoka *et al*, 2006) cancer were correlated with a poor prognosis (Wolf *et al*, 2005; Hiraoka *et al*, 2006). In particular, multivariate analysis in pancreatic cancer (Hiraoka *et al*, 2006) showed that the prevalence of Foxp3^+^ T regs was an independent prognostic factor. On the contrary, it has been reported that the clinical outcome was not dependent on the prevalence of Foxp3^+^ T regs in TILs in renal cell carcinoma (Siddiqui *et al*, 2007). Thus, in human malignancies, it remains controversial as to whether infiltrating T regs, in particular Foxp3^+^ T regs, are related to the clinical outcome.

In the current study, we analysed the prevalence of Foxp3^+^ cells in gastric cancer by immunohistochemistry. The prevalence of Foxp3^+^ cells was significantly increased in the tumour lesion compared to normal gastric tissue. Moreover, the population of Foxp3^+^ cells in stage IV was significantly larger than those in stage I. However, the prevalence of Foxp3^+^ cells is not significantly associated with the overall survival of patients with gastric cancer, in contrast with previous reports on ovarian carcinoma (Wolf *et al*, 2005) and pancreatic cancer (Hiraoka *et al*, 2006). The present study is, to our knowledge, the first report describing the prevalence of Foxp3^+^ T regs related to the prognosis in gastric cancer.

With regards to gastric mucosa, there are several reports describing the presence of *Helicobacter pylori* infection-associated Foxp3^+^ T regs (Lundgren *et al*, 2005; Enarsson *et al*, 2006). Since, in the present cohort with gastric cancer, there was no information on *H. pylori* infection, it is impossible to evaluate the influence of this infection. Thus, the presence of *H. pylori* infection may lead to complications in the evaluation of Foxp3^+^ T regs in tumoral immunity of gastric cancer.

Of note, we found that the patients with a predominant localisation of Foxp3^+^ cells in the peri-tumoral region had a better prognosis than those showing a diffuse localisation of Foxp3^+^ cells. Since the precise mechanisms inducing and expanding T regs remain unclear, the biological difference between the peri-tumoral and diffuse localisation of Foxp3^+^ cells is under investigation. It is possible to presume that a tumour-related factor may induce an accumulation of Foxp3^+^ T regs in the peri-tumoral region at the early stage of gastric cancer, since stage I patients with a peri-tumoral localisation pattern were significantly more frequent. For example, the cancer–stromal reaction including the migration factors for Foxp3^+^ T regs may differ within tumours, leading to the difference in the localisation pattern of Foxp3^+^ T regs. That is, the migration factors for Foxp3^+^ T regs were mainly produced in the peri-tumoral region as a result of the tumour–stromal reaction at the early stage of gastric cancer.

In conclusion, the prevalence of Foxp3^+^ cells is not significantly associated with the overall survival of patients with gastric cancer. However, the patients with a predominant localisation of Foxp3^+^ cells in the peri-tumoral region had a better prognosis than those showing the diffuse localisation of Foxp3^+^ cells. A better understanding of the underlying mechanism of T reg regulation or a strategy for controlling T regs may lead to a novel therapeutic strategy for gastric cancer.

## Figures and Tables

**Table 1:** Patient and tumour characteristics (*n*=80)

Age (years, mean±s.d.)	61±13
	
*Gender*	
Male : female	56 : 24
	
*Tumour size (mm)*	
Mean (±s.d.)	54.1±42.3
	
*Tumour* a	
pT1	31
pT2	28
pT3	15
pT4	6
	
*Lymph node metastasis* a	
pN0	41
pN1	21
pN2	9
pN3	9
	
*Histological classification*	
Intestinal type	41
Diffuse type	39
	
*Stage* a	
I A	30
I B	12
II	12
III A	11
III B	4
IV	11

a Tumour, lymph node metastasis, and stage according to the Japanese Classification of Gastric Carcinoma (Japanese Gastric Cancer Association, 1998).

**Table 2:**
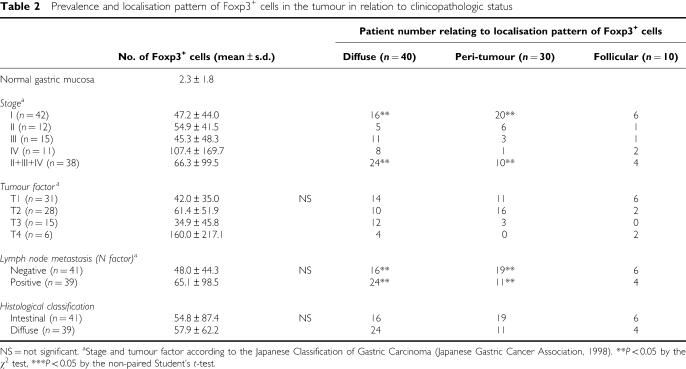
Prevalence and localisation pattern of Foxp3^+^ cells in the tumour in relation to clinicopathologic status

**Table 3:** Univariate and multivariate analysis of the patients

		**Univariate analysis**		**Multivariate analysis**	
		**Overall survival**		**Overall survival**	
**Variables**	**Categories**	**HR (95% CI)**	***P***	**HR (95% CI)**	***P***
Age (years)	<60 (*vs* ⩾60)	1.64 (0.71–3.79)	0.25		
Gender	Male (*vs* female)	0.42 (0.18–0.98)	0.45		
Tumour size (mm)	⩾40 (*vs* <40)	7.94 (1.84–34.48)	0.0055	0.94 (0.063–14.08)	0.96
Tumour^a^	pT2+pT3+pT4 (*vs* pT1)	3.64 (1.23–10.75)	<0.0001	0.67 (0.16–2.81)	0.59
Lymph node metastasis^a^	pN1+pN2+pN3 (*vs* pN0)	14.29 (3.32–62.50)	0.0003	1.44 (0.13–15.87)	0.76
Stage^a^	Stages III+IV (*vs* stages I+II)	8.47 (3.30–21.74)	<0.0001	0.67 (0.15–3.08)	0.61
Population of Foxp3^+^ cells^b^	High (*vs* low)	0.85 (0.37–1.97)	0.70		
Localisation pattern	Diffuse (*vs* peri-tumour)	4.65 (1.35–15.96)	0.0147	1.98 (0.42–9.39)	0.39
Infiltration type^a^	INF-*γ* (*vs* INF-*α*+INF-*β*)	5.56 (2.17–14.35)	0.0004	2.70 (0.67–10.94)	0.16
Histological classification	Diffuse (*vs* intestinal)	3.13 (1.22–8.00)	0.0173	1.75 (0.33–9.17)	0.51
Lymphatic invasion	Positive (*vs* negative)	23.13 (3.09–173.09)	0.0022	8.44 (0.53–133.92)	0.13
Vascular invasion	Positive (*vs* negative)	9.43 (3.40–26.32)	<0.0001	5.41 (1.10–26.32)	0.037

95% CI=95% confidence interval; HR=hazard ratio; INF=infilteration.

a Tumour, lymph node metastasis, stage, and infiltration type according to the Japanese Classification of Gastric Carcinoma (Japanese Gastric Cancer Association, 1998).

b Foxp3^+^-high and Foxp3^+^-low groups were classified by the median value of the total population of Foxp3^+^ cells (median=34.5, range=2–525).
